# 

**Published:** 1823-12

**Authors:** 


### MONTHLY CATALOGUE OF MEDICAL BOOKS.

*** *It having been hinted to us that gentlemen, sending copies of their Works, prefer having their titles given at length, it is our intention, in future, to comply with this suggestion: but it is to be observed, that no books can be entered on this List except those sent to us for the purpose;—finding, in the lists hitherto transmitted, that the names of works have frequently been given as published, which have not appeared for weeks, or even months after.*

Suggestions on the Use of Mercurial Friction on and under the Right Breast, if possible, to palliate the Evils of the Fever of Hot Countries. By WILLIAM FORBES, A.M. Surgeon, Manchester, Jamaica.—Jamaica, 1823.

Outlines of Character: consisting of the Great Character—the English Character—Characteristic Classes, in relation to Happiness—the Gentleman—External Indications of Character—Craniology—the Poet—the Orator—Literary Character—the Periodical Critic—the Man of Genius, &c. By ROBERT MAUGHAM. Second Edition.—pp. 306. London, 1823.

Medicinisch-Chirurgische Zeitung fortgesebt. Von Dr. JOHANN NEPOMUCK EHRHART. Dutter Band.—1823.

A Statement of the early Symptoms which lead to the Disease termed "Water in the Brain;" with Observations on the Necessity of a watchful Attention to them, and on the fatal Consequences of their Neglect. By G. D. YEATS, M.D. F.R.S.; Fellow of the Royal College of Physicians, London, of the Royal Medical Society, Edinburgh; Honorary Member of the Dublin Society; Physician to H. R. H. the Duke of Cumberland, to his Grace the Duke of Bedford; one of the Physicians to the Asylum for Female Orphans; and late Physician to the Lunatic Asylum and Infirmary of the County and Town of Bedford. Second Edition, considerably enlarged.—pp. 164. London, 1823.

Lectures on the general Structure of the Human Body, and on the Anatomy and Functions of the Skin; delivered before the Royal College of Surgeons in London, in the course of 1823. By THOMAS CHEVALIER, F.R.S. F.S.A. F.L.S. and F.H.S.; Surgeon Extraordinary to the King, and Professor of Anatomy and Surgery to the College.—pp. 277. London, 1823.

Deutsches Archiv für die Physiologie, &c. &c. Von J. F. MECKEL. Achter Band. Zweiter Heft.—1823.

De Nervi Sympathetici Humani, fabrica, usu, et morbis; Commentatio-Anatomico, Physiologico, Pathologica, Tabulis œneis, et Lithographicis illustrata. Auctore JOH. FRID. LOBSTEIN, Medecinæ Clinices et Anatomiæ Pathologicæ in Facultate Medica, Argentoratensi Professore, Nosocomii civium Medico Obstetrico primario, plurum Societat. Medicarum sodali.—pp. 174. Parisiis, 1823.

Philadelphia Journal of the Medical and Physical Sciences, supported by an Association of Physicians, and edited by N. CHAPMAN, M.D. &c.—Philadelphia, August 1823.

An Engraved Representation of the Anatomy of the Human Ear, exhibiting in one view the external and internal parts of that Organ *in situ*, accompanied with a Plate of Outlines and References, with copious explanations, to which are added, Surgical Remarks on introducing the Probe and Catheter into the Eustachian Tube by the Nostril,—on the operation of puncturing the Membrana Tympani,—and a Synoptical Table of the Diseases of the Ear, their Classification, Seat, Symptoms, Cause, and Treatment: the whole designed as a Guide to Acoustic Surgery. By THOMAS BUCHANAN, C.M. Licentiate of the University of Glasgow, Corresponding Member of the Phrenological Society of Edinburgh, and Surgeon to the Hull Dispensary for Diseases of the Eye and Ear.—pp. 38. Hull, 1823.

Observations on Prison Discipline, exemplified by the Tread-mill and Dietary adopted in the Nottinghamshire House of Correction at Southwell. By BENJAMIN HUTCHINSON, Surgeon to the Establishment, Fellow of the Royal College of Surgeons, and of the Medical Society of London.—Newark. Sold by Baldwin and Co. Paternoster-row. 1

### NOTICE TO CORRESPONDENTS.

*We beg to state, that the Paper on* Delirium Ebriositatis, *which appeared in our last Number, was not taken from the Edinburgh Medical and Surgical Journal; but that the* original MS. *was sent to us, and, but for press of matter, would have been published sooner. It had passed the press before the Edinburgh Journal came to hand.*

*The Papers of* MR. A. C. HUTCHISON, MR. R. BROWN, MR. RENWICK, *and* MR. AVERILL, *have been received, and shall meet with the earliest insertion.*

